# Drivers of Hirola Antelope Diet Selection in Natural and Managed Habitat in Eastern Kenya

**DOI:** 10.1002/ece3.72980

**Published:** 2026-01-22

**Authors:** Abdullahi H. Ali, S. Kivai

**Affiliations:** ^1^ Hirola Conservation Program Garissa Kenya; ^2^ National Museums of Kenya Nairobi Kenya

**Keywords:** diet selection, forage quality, habitat management, hirola, restoration, species recovery

## Abstract

Diet selection, referring to the food that is ingested by an animal along with its nutritional composition, is essential for animal fitness, survival and behavior. The hirola ( *Beatragus hunteri* ), with ~500 individuals remaining, is classified as Critically Endangered by IUCN and with range degradation from tree encroachment implicated in their decline. For effective hirola recovery, there has to be a clear understanding of habitat‐specific forage quality, prior to planned re‐introduction efforts from managed into natural habitats. We studied populations in natural and managed breeding habitat in eastern Kenya to assess the dietary selection of hirola. Hirola consumed a total of 17 species with *Chloris virgata* being the most preferred grass, while *Commelina benghalensis* and *Commelina diffusa* were the most preferred forbs. *Cyperus kilimandscharicus* was the only moderately preferred sedge. We then analyzed nutrient content for 14 variables, including crude protein (CP), fiber fractions, in vitro dry matter digestibility (IVDMD), and key minerals. Forage in natural habitats was more closely associated with higher levels of phosphorus (P), magnesium (Mg), and IVDMD, while managed habitats were characterized by elevated sodium (Na), potassium (K), and CP levels. Seasonal differences were also detected, particularly in calcium (Ca) and Mg concentrations. Our results reveal substantial variation in plant chemical composition, forage quality and diversity between habitats, necessitating adaptive foraging strategies. Future habitat restoration programs should focus on monitoring and reseeding preferred forage species and the provisioning of supplementary diets with appropriate chemicals to support newly released herds or in the breeding facility.

## Graphical abstract

Hirola ( *Beatragus hunteri* ) populations in eastern Kenya consumed 17 forage species, favoring the grass *Chloris virgata* and the forbs *Commelina benghalensis* and *C. diffusa* . Nutrient analyses showed that natural habitats offered higher phosphorus, magnesium, and digestibility, whereas managed sites provided more sodium, potassium, and crude protein, with notable seasonal shifts in calcium and magnesium. These patterns highlight the need for habitat‐specific reseeding of preferred plants and targeted mineral supplementation during future restoration and reintroduction efforts.

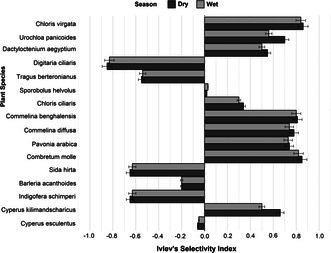

## 1 Introduction

African savannas are shaped by a complex interplay of environmental factors that influence growth, flowering, and fruiting patterns across different ecosystems (Mishra and Young 2020). These variations have significant ecological consequences, particularly affecting the behavior and distribution of herbivores and other wildlife dependent on plant resources. Understanding these plant characteristics is critical for conservation and ecosystem management, as they are controlled by a combination of weather and climatic patterns, including rainfall and temperature fluctuations, soil nutrient availability, and other environmental variables. The characteristic seasonal changes in savanna climates alternating between distinct wet and dry periods strongly influence plant nutritive dynamics (Ravhuhali et al. 2022). During the wet season, which typically occurs in the summer months, plants experience increased growth and flowering due to the abundance of water and nutrients. This phase is vital for many species, allowing them to complete their reproductive cycles and store nutrients essential for survival in the upcoming dry season. Conversely, the dry season results in reduced plant growth and flowering as water becomes scarce, forcing plants to conserve energy and resources (Sankaran et al. 2005; Wigley et al. 2020). For instance, in the Serengeti ecosystem of East Africa, the wet season (November to May) triggers the proliferation of nutrient‐rich grasses such as *Themeda triandra* and *Cynodon dactylon* , which serve as key forage for herbivores like wildebeests and zebras (Sinclair et al. 2003). These seasonal plant variations, in turn, influence herbivore movements and distribution across landscapes (Holdo et al. 2009). Similarly, in Kruger National Park, South Africa, and other major conservation areas in East Africa, enhanced rainfall during the wet season spurs the growth of tree species such as *Acacia nigrescens* and *Sclerocarya birrea* , alongside various grasses and herbs, which are vital food sources for a diverse range of herbivores (Smit et al. 2007).

The dietary composition of herbivorous mammals is closely tied to their nutritional ecology (Felton et al. 2020). The ability of herbivores to optimize their macronutrient intake directly affects their health, reproductive success, and population dynamics. Studies have established strong correlations between plant food quality and herbivore population growth rates (Sibly and Hone 2002; Maklakov et al. 2008). In response to spatial and temporal fluctuations in forage quality, ungulates exhibit dietary shifts (Owen‐Smith 2002), often expanding their diets to include less‐preferred species when high‐quality forage is scarce, such as during droughts or periods of increased competition (Stewart et al. 2011). However, dietary contractions can occur due to habitat degradation, climatic stress, or heightened competition (Parker et al. 2009). In particular, arid and semi‐arid environments exacerbate the seasonal loss of leaves from drought‐deciduous vegetation, leading to periods of protein deficiency for herbivores (Alderman et al. 1989; Cain et al. 2008).

Habitat management and bare land restoration play a crucial role in influencing forage availability and herbivore feeding behavior, particularly in protected areas such as sanctuaries and conservancies. These managed landscapes often implement controlled grazing, habitat restoration, and predator management, all of which can affect the distribution and quality of forage resources. In some cases, sanctuaries with restricted herbivore movement and artificial water points may lead to localized overgrazing and nutritional deficiencies (Hayward and Kerley 2009). Conversely, well‐managed community conservancies can promote habitat heterogeneity, ensuring diverse forage availability across seasons and improving overall herbivore fitness (Scholte et al. 2007).

Ungulate forage selection is largely driven by nutritional limitations, particularly protein, energy, and mineral availability (Gou et al. 2024). In temperate regions, species such as the white‐tailed deer ( *Odocoileus virginianus* ) prioritize energy‐rich forage over protein‐dense plants (Dumont et al. 2005). In contrast, in tropical and subtropical environments, ungulates often experience protein limitations (Jarman and Sinclair 1979; Prins and Beekman 1989; Cain et al. 2017). In arid regions where surface water is limited, herbivores may select plants based on moisture content rather than nutritional value, such as Opuntia species, which have high water content but low protein levels (Alderman et al. 1989; Cain et al. 2008).

The hirola antelope ( *Beatragus hunteri* ), endemic to East Africa and classified as Critically Endangered, faces a particularly urgent conservation challenge due to forage availability. With an estimated population of fewer than 500 individuals, hirola numbers continue to decline due to range degradation over the past 3–4 decades (Andanje 2002; Ali et al. 2017; Ali et al. 2019). Hirola are uniquely adapted to arid environments with annual rainfall ranging from 300 to 600 mm. They prefer open to lightly bushed grasslands and wooded savannas with scattered trees and low‐stature shrubs (Andanje 2002). The highest population densities have been recorded in well‐drained, white sandy soils, particularly in *Digitaria milanjiana* ‐, *Chloris mossambicensis*‐, and *Dobera glabra*‐dominated wooded‐bushed grasslands. Due to its small population, in the year 2012, a breeding founder population of 48 individuals was moved into a fenced predator‐free managed habitat for conservation breeding and to serve as a source of future reintroductions. As a strict grazer, hirola represents a challenge to the conservation breeding facility due to its sensitivity to nutritional management and recurrent droughts. As a specialist feeder, hirola primarily relies on select grass species, making its survival highly dependent on the availability and nutritional quality of these preferred forage resources. Since forage quality is a key determinant of population recovery in endangered species, a deeper understanding of the relationship between dietary resources and hirola population dynamics is crucial. Although prior research on hirola has shown habitat preferences, population dynamics, and range degradation (Ali et al. 2017, 2019; Andanje 2002), no study has directly linked field‐based feeding observations with thorough laboratory analyses of forage nutritional quality in both natural and managed breeding habitats. This gap is crucial because effective reintroduction programs rely on knowing not only what hirola eat but also the nutritional adequacy of available forage at the release sites. Further, while other studies have explored the nutrition of large herbivores during times of food scarcity in a single breeding facility, our study examines forage selection and quality across populations under different management regimes, offering crucial baseline information for assessing the nutritional preparedness of reintroduction sites (Hejcmanová et al. 2019). Our study therefore aimed at assessing the dietary selection of hirola antelope (i.e., composition, forage quality, and seasonal variations) in populations in natural and managed breeding habitats in eastern Kenya, specifically to: (1) quantify dietary preferences using selectivity indices, (2) characterize habitat‐specific differences in forage nutritional composition, and (3) evaluate seasonal nutritional variation. These findings contribute to a broader understanding of the interplay between environmental variables, forage selection, and population regulation, and will be critical in guiding future hirola reintroduction efforts, ensuring release sites can adequately support hirola dietary requirements and survival.

## 2 Methodology

### 2.1 Study Site

**FIGURE 1:**
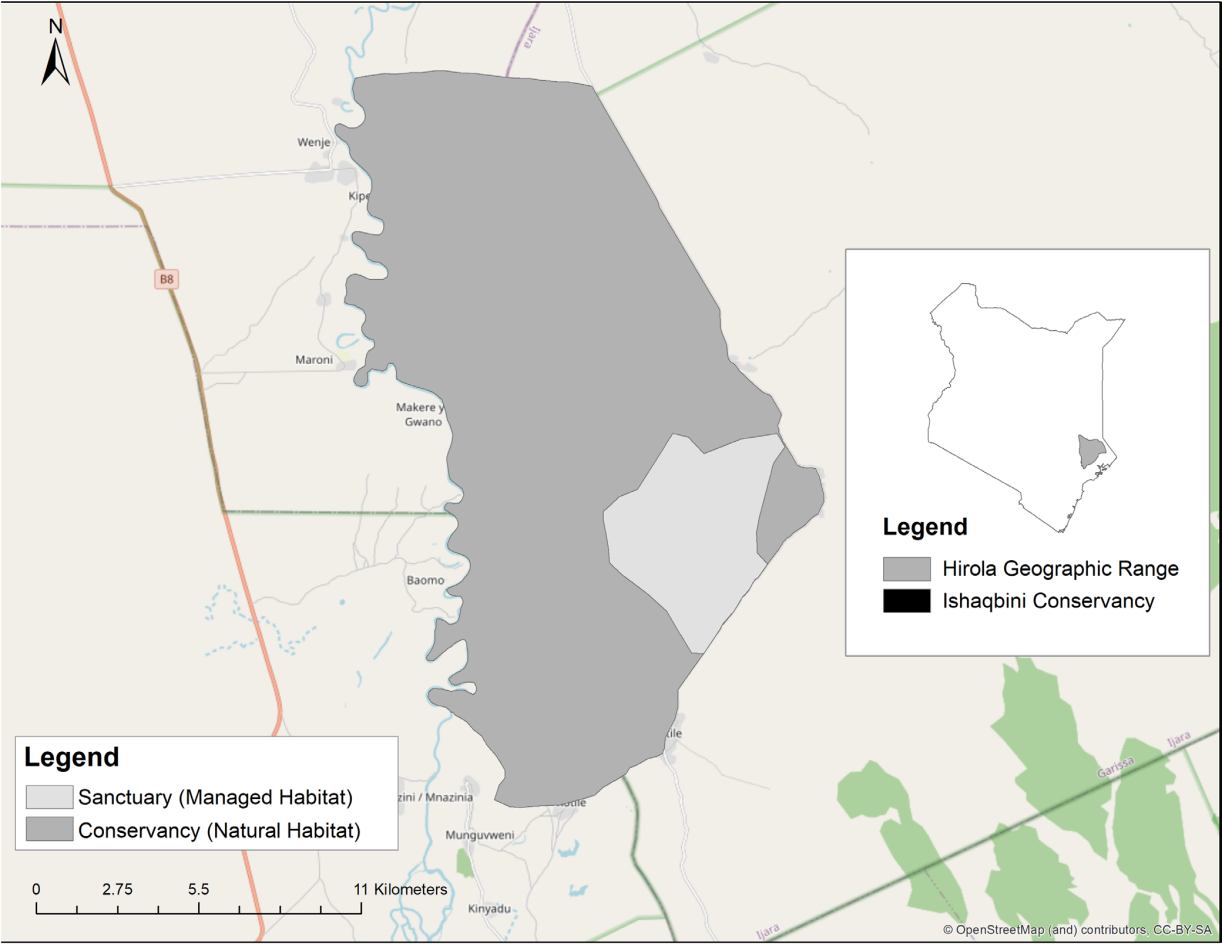
Map of study sites showing Ishaqbini Conservancy (natural habitat) and Sanctuary (managed habitat) in Garissa County, Kenya. The map highlights their relative positions and that of the hirola's geographic range within Kenya and key geographical features. Ishaqbini Conservancy supports natural wildlife, while the fenced Ishaqbini Sanctuary focuses on breeding the hirola antelope.

Our study sites comprise two different settings; (1) hirola managed habitat (Sanctuary): A predator‐proof sanctuary embedded within the conservancy and covering 27 km^2^. The sanctuary was established in 2012 and is enclosed by a 2.5 m high electric fence. Daily fence patrols are conducted and there are minimal human disturbance within the sanctuary. Large carnivores were removed prior to hirola translocation, and permanent water troughs were established. The sanctuary currently supports a managed hirola population alongside approximately 15 elephants introduced to control woody encroachment and a limited number of cattle (~30 heads) used for tick control and (2) natural habitat (Conservancy): Encompasses the sanctuary and covers approximately 240 km^2^ on the eastern bank of the Tana River in Kenya. It was established in 2005 by Terra Nuova and in contrast to the sanctuary, the conservancy contains a full complement of native predators and represents a semi‐natural rangeland system. Livestock grazing has been reduced since 2008 to improve habitat quality, although wildlife–predator interactions occur naturally. Multiple hirola groups use the conservancy seasonally (Figure 1). These two sites fall within the larger hirola's native range under Fafi sub‐county (latitude: 0°25′23.26″ S, longitude: 40°13′46.42″ E) and Ijara sub‐county (latitude: 1°36′33.95″ S, Longitude: 40°32′35.43″ E) in Garissa County, Kenya. These areas are classified as Arid Lands with an average rainfall of between 350 and 550 mm and considerably high temperatures that can reach up to 40°C during the dry seasons. The region's vegetation is mostly shrubby with thorny bush donning patches of diminishing semi‐arid grasslands. The common shrubs in this habitat include *Acacia bussei*, *Boscia coriacea*, *Cadaba heterotricha*, *Combretum aculeatum*, *Commiphora* spp., *Terminalia orbicularis*, *Acacia tortilis* , *Caesalpinia trothae*, *Caucanthus albidus*, *Cassia longiracemosa*, *Ehretia teitensis* and *Thylachium thomasii*. The dominant grass species include; *Brachiaria dejlexa*, *Brachiaria leersioides*, *Cenchrus ciliaris* , *Digitaria macroblephara*, *Digitaria rivae*, *Latipes senegalensis*, *Panicum maximum* , *Aristida adscensionis* , *Chloris roxburghiana*, *Tetrapogon tenellus* and *Sporobolus helvolus* (Andanje 2002). The managed habitat is fenced and access by livestock is limited, while the natural area is open and accessible by both wildlife and livestock. The managed habitat experiences less pressure from overgrazing by livestock compared to the latter. Therefore, vegetation in this habitat is seemingly healthier due to decreased grazing pressure from livestock regeneration rate and better range conditions than in the natural habitat.

In this region, wildlife and people have long shared the landscape, shaping an intricate balance between conservation and pastoralism. The local Somali pastoralist communities primarily herd camels ( *Camelus dromedarius* ), cattle ( *Bos indicus* ), goats ( *Capra hircus* ), and sheep ( *Ovis aries* ), relying on these animals for their livelihood (Ali et al. 2017). Among the diverse wildlife that roam these lands is the critically endangered hirola ( *Beatragus hunteri* ), living alongside other herbivores such as the reticulated giraffe ( *Giraffa camelopardalis reticulata* ), African buffalo ( *Syncerus caffer* ), gerenuk ( *Litocranius walleri* ), waterbuck ( *Kobus ellipsiprymnus* ), lesser kudu ( *Tragelaphus imberbis* ), plains zebra ( *Equus burchellii* ), topi ( *Damaliscus lunatus* ), Kirk's dik‐dik ( *Madoqua kirkii* ), desert warthog ( *Phacochoerus aethiopicus* ), and Somali bush baby ( *Galago gallarum* ).

Predators are also a key part of this ecosystem, with lions ( *Panthera leo* ), leopards ( *Panthera pardus* ), spotted hyenas ( *Crocuta crocuta* ), cheetahs ( *Acinonyx jubatus* ), and African wild dogs ( *Lycaon pictus* ) playing vital roles in maintaining ecological balance. The region is also home to a diverse bird population, including Clarke's weaver ( *Ploceus golandi* ), the hooded vulture ( *Necrosyrtes monachus* ), the saddle‐billed stork ( *Ephippiorhynchus senegalensis* ), the eastern violet‐backed sunbird ( *Anthreptes orientalis* ), the white‐throated bee‐eater ( *Merops albicollis* ), and the crested francolin (*Ortygornis sephaena*; Njoroge et al. 2008).

### 2.2 Data Collection

#### 2.2.1 Feeding Behavior Observations

We conducted the behavioral observations using the focal animal sampling approach (Altmann 1974). We carried out data collection between March 2021 and March 2022, where a single hirola antelope was observed continuously for 10 min with resting intervals of 5 min. We recorded focal observations in the morning hours from 0600 to 1130 h and late afternoon from 1600 to 1800 h, when the hirolas were actively feeding. We observed the hirola individuals on a rotational basis and recorded the sex, age class, and group size. We made efforts to sample at least every individual before repeat samplings were done. During the hirola focal observations, we recorded data on plant food species eaten, life form, the plant parts eaten, and the habitat location where feeding activity occurred (Altmann 1974). We defined individual feeding or ingestion rate as the average number of mouthfuls or bites of food ingested per 60 s of observation during the feeding bouts. To obtain the average feeding rate for different foods, at least 10 observations per food item were recorded.

#### 2.2.2 Grass Nutritional Sample Collection

We collected approximately 76 food samples consisting of various species of grasses, sedges, herbs, and shrubs. We collected the samples during both the wet (*N* = 30 samples) and dry seasons (*N* = 46). During the focal observations, we ensured that each food sample weighed at least 250 g when wet to ensure a minimum of 50 g of dry weight per sample. The food samples were then dried in the field using a food dehydrator set at 55°C. To determine the moisture content, we recorded the wet weight before placing the samples in the dehydrator and measured the constant dry weight after complete dryness, which was done after three subsequent measurements taken every 5 h during the drying process. Furthermore, to estimate the food ingestion rates, we determined the average fresh weight (g) of an equivalent bite size or mouthful for each food item consumed and repeated the process 10 times to account for natural variation in bite size, reduce measurement error, and improve statistical reliability. The sampling effort and the distribution of focal observations and forage samples across habitats and seasons are summarized in Table 1.

**TABLE 1:** Summary of focal observation effort and forage sampling across managed and natural habitats.

Parameter	Natural habitat	Managed habitat	Total
Focal animals observed	28	32	60
Total observation hours	142	156	298
Wet season samples (*n*)	14	16	30
Dry season samples (*n*)	22	24	46
Total forage samples	36	40	76

### 2.3 Data Analysis

We analyzed a total of 76 food samples from hirola feeding sites in the natural and modified habitats for chemical composition at the Animal Nutrition Laboratory of the upper Kabete Campus of the University of Nairobi in Kenya. The plant foods underwent comprehensive analysis for key macro‐ and micronutrients, including fiber (Neutral Detergent Fiber, Acid Detergent Fiber, and Lignin), lipids (Ether Extract, EE), Crude Protein (CP), and Total Non‐structural Carbohydrates (TNC). The micronutrients included: copper (Cu), iron (Fe), zinc (Zn), potassium (K), sodium (Na), manganese (Mn), magnesium (Mg), calcium (Ca), and phosphorus (P). Additionally, dry matter, moisture content, and ash were determined for all samples. The three fiber components in the food samples were analyzed using an A200 ANKOM fiber analyzer. Protein content was determined by first analyzing total nitrogen (N) in the food using a Leco FP‐528 combustion analyzer, then multiplying the total nitrogen by a constant of 6.25 to obtain the crude protein content. Lipid content was determined through Ether Extract (EE) using standard procedures (Kivai 2018). The nutritional content of the hirola foods was reported as a percentage of the dry matter of the food organic matter (Conklin‐Brittain et al. 2006).

To quantify the level of preference or avoidance for these food types, we employed Ivlev's Selectivity Index, a metric that helps determine the degree to which an animal favors certain food sources over others based on their availability (Jacobs 1974). We quantified plant species availability for calculating Ivlev's Selectivity Index using quadrat‐based vegetation surveys. In each habitat and season, we placed 1 m × 1 m quadrats along transects covering both feeding and non‐feeding areas. Within each quadrat, we recorded all plant species present. We then calculated availability as encounter frequency, defined as the proportion of quadrats in which each species occurred. We used these frequency‐based availability estimates to compute Ivlev's Selectivity Index by comparing the proportion of each species consumed with its proportional availability. We used *t*‐tests to compare the Shannon–Wiener Diversity Index (H′), a commonly used measure of biodiversity that quantifies the diversity of a community. We used Analysis of Variance to compare means of macro‐ and micronutrient content among different areas and seasons. Further, we employed Chi‐squared tests to analyze the proportions of various food types consumed by hirola. This analysis provided insights into significant differences in species preference. Prior to PCA analysis, we standardized all 14 nutritional variables using *z*‐score transformation (mean = 0, SD = 1) to ensure comparability across nutrients with different measurement units and scales. This standardization prevented variables with larger absolute values (e.g., %DM, %NDF) from disproportionately influencing the ordination relative to trace minerals measured in parts per million. Finally, we used Principal Component Analysis (PCA) to analyze forage nutritional composition from two habitat types within the hirola's range: Natural and Managed. The data set comprised nutrient content values from a total of 76 samples (36 from Natural habitat and 40 from Managed habitat) collected across sites classified as Natural (Ishaqbini Conservancy) and Managed (Ishaqbini Sanctuary). Each sample included measures of 14 nutritional variables commonly used to assess forage quality: dry matter (%DM), ash (%ASH/DM), ether extract (%EE/DM), crude protein (%CP/DM), acid detergent fiber (%ADF/DM), acid detergent lignin (%ADL/DM), total non‐structural carbohydrates (TNC/DM), in vitro dry matter digestibility (%IVDMD), and macro‐ and micronutrients including Na, K, Mg, Ca, P, and Mn. PCA was performed in RStudio v2024.12.1 using the PCA() function from the FactoMineR R package, and visualized using fviz_pca_biplot() from factoextra. Prior to analysis, we assessed all continuous response variables for normality using Shapiro–Wilk tests and by visual inspection of residual plots. We evaluated homogeneity of variances using Levene's tests. Where assumptions were violated, we log‐ or square‐root transformed variables prior to analysis. We then assessed differences among groups using *t*‐tests or one‐way ANOVA as appropriate. When ANOVA results were significant, we conducted post hoc pairwise comparisons using Tukey's honestly significant difference (HSD) test to control for multiple comparisons. We conducted all statistical analyses in RStudio v2024.12.1, with significance assessed at *α* = 0.05.

## 3 Results

### 3.1 Plant Species Utilized by Hirola Antelopes

From the 76 samples analyzed, we identified 17 plant species consumed by hirola, belonging to seven families: Acanthaceae, Combretaceae, Commelinaceae, Cyperaceae, Fabaceae, Malvaceae, and Poaceae. The Poaceae family had the highest representation, including *Sporobolus helvolus*, *Chloris virgata* , *Eragrostis biflora*, *Tragus berteronianus* , *Digitaria ciliaris* , *Dactyloctenium aegyptium* , and *Chloris ciliaris*. Other notable species included *Commelina benghalensis* , *Commelina diffusa* , *Sida hirta* , *Pavonia arabica*, *Cyperus esculentus* , *Cyperus kilimandscharicus*, *Indigofera schimperi* , *Combretum molle*, and *Barleria acanthoides* (Table S1). We found that grasses were the most abundant plant form, with the highest availability recorded in the managed habitat during the wet season (63.3%) and the lowest in the natural habitat during the dry season (43.5%). Herbs were more prevalent in the natural habitat (33.8% in the dry season, 25.3% in the wet season), while in the managed habitat, they declined significantly during the wet season (13.3%). Forbs and sedges remained stable across habitats and seasons (Figure S1). No significant variation was observed in plant form distribution (*χ* ^2^ = 14.42, *p* = 0.1080). Our analysis showed a Shannon–Weiner diversity index of 2.59 (evenness 0.71). The managed habitat had a higher diversity index (2.64) than the natural habitat (2.31), with the difference being statistically significant (*t* = 2.63, *p* = 0.0121). *Sporobolus helvolus* (18.4%) and *Chloris virgata* (13.2%) were the most consumed, while other species contributed less, with statistically significant differences (*χ* ^2^ = 79.69, df = 18, *p* < 0.0001). We found no significant variations in consumption based on season, sex or location (*p* > 0.05) as summarized in Figure S2.

### 3.2 Selectivity Index for Hirola Food Types

**FIGURE 2:**
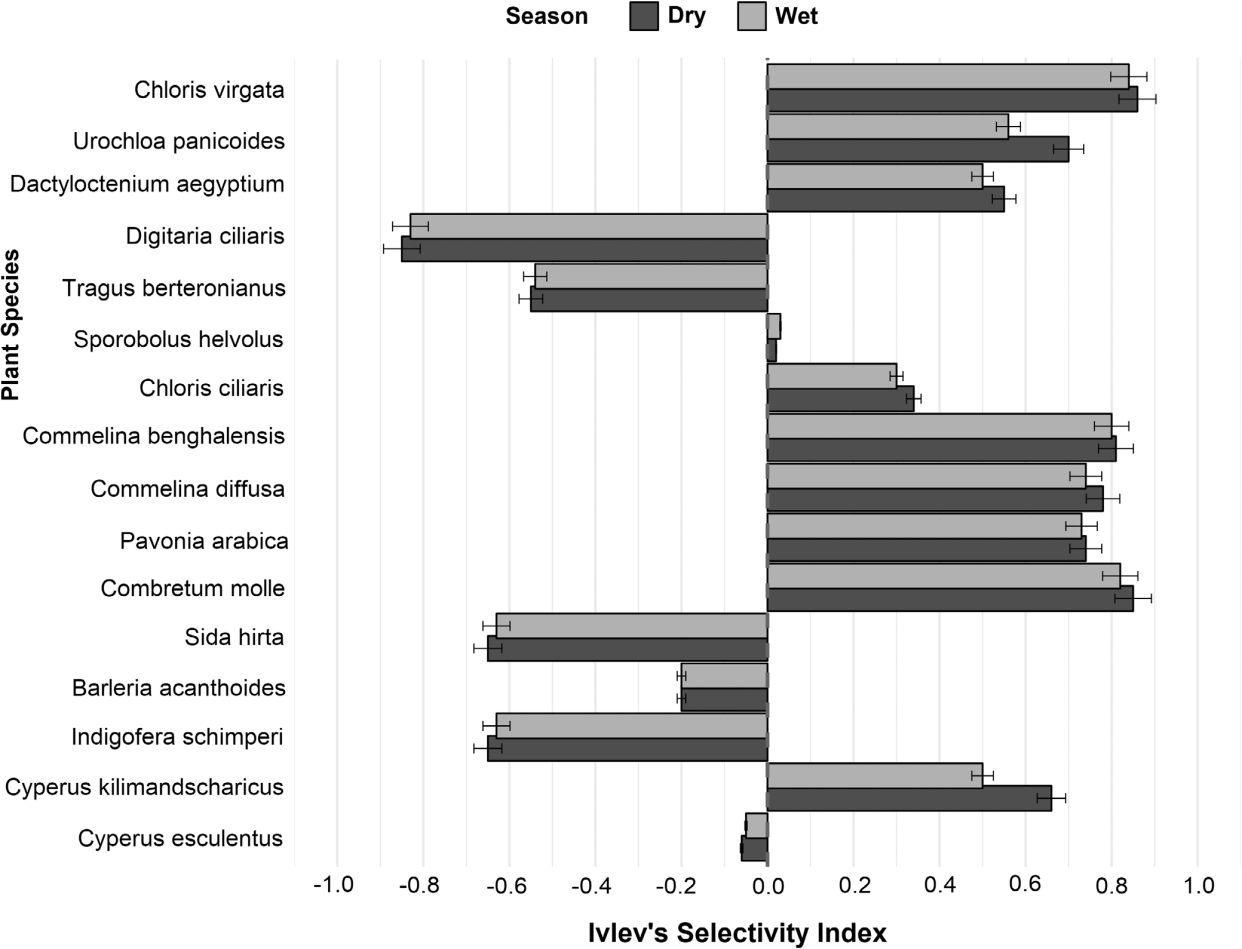
Ivlev's Selectivity Index for hirola food types across dry and wet seasons (*n* = 46 and 30 samples, respectively). Positive values indicate preference, while negative values represent avoidance.

Hirola's Ivlev's Selectivity Index revealed variations in plant selection across different seasons. Hirola exhibited a higher preference for certain forbs and grasses, while some plant species were consistently avoided (Figure 2). However, the location (whether in a natural or managed habitat) did not influence forage selectivity (*p* > 0.05). Among grasses, *Chloris virgata* was the most preferred species (0.86; 0.84), followed by *Urochloa panicoides* (0.70; 0.56) and *Dactyloctenium aegyptium* (0.55; 0.50) in dry and wet seasons respectively, suggesting their importance as key dietary components. However, *Digitaria ciliaris* (−0.85; −0.83) and *Tragus berteronianus* (−0.55; −0.54) were strongly avoided.

*Sporobolus helvolus* (18.4%) and *Chloris virgata* (13.2%) were the most frequently consumed species based on feeding observations, with significant differences among species (*χ* ^2^ = 79.69, df = 18, *p* < 0.0001). However, high consumption does not necessarily indicate strong dietary preference. Ivlev's Selectivity Index (Figure 2) shows that while *Chloris virgata* was highly preferred, *Sporobolus helvolus* exhibited neutral to low selectivity, reflecting proportional consumption relative to its high availability in the habitat rather than active selection. Such discrepancies between proportional intake and selectivity indices are well documented in herbivore foraging studies, where abundant species dominate diets through encounter rates rather than active preference (Cain et al. 2017).

Ivlev's Selectivity Index revealed clear patterns of preference and avoidance among forage species (Figure 2). For shrubs, *Combretum molle* (dry season: 0.85; wet season: 0.82) was highly preferred in both seasons. Among forbs, *Commelina benghalensis* (0.81; 0.80) and *Commelina diffusa* (0.78; 0.74) were highly preferred in both seasons, indicating their importance in hirola's diet. Similarly, *Pavonia arabica* showed moderate preference (0.74; 0.73). In contrast, *Sida hirta* was avoided during both seasons, with negative selectivity values (−0.65; −0.63). For herbs, *Barleria acanthoides* (−0.20; −0.20) and *Indigofera schimperi* (−0.65; −0.63) were avoided, showing no seasonal variation in preference. Among sedges, *Cyperus kilimandscharicus* was moderately preferred (0.66; 0.50), whereas *Cyperus esculentus* was generally avoided (−0.06; −0.05), illustrated in Figure 2.

### 3.3 Comparison of Macronutrients in Plant Food Species

The dry matter content ranged from 92.9% ± 0.2% in *Digitaria ciliaris* to 98.5% ± 0.1% in *Tragus berteronianus* . Ash content varied, with *Cyperus kilimandscharicus* having the highest (21.1% ± 0.3%) and *Tragus berteronianus* the lowest (7.9% ± 0.2%). Ether extract was generally low (< 5%), except in *Pavonia arabica* (9.9% ± 0.4%) and *Tragus berteronianus* (9.3% ± 0.3%). Crude protein content ranged from 11.5% ± 0.2% in *Dactyloctenium aegyptium* to 28.8% ± 0.5% in *Digitaria ciliaris* . Fiber fractions varied, with Neutral Detergent Fiber (NDF) ranging from 34.2% ± 0.6% in *Indigofera schimperi* to 76.0% ± 0.8% in *Sporobolus helvolus*, Acid Detergent Fiber (ADF) from 22.0% ± 0.5% in *Indigofera schimperi* to 54.6% ± 0.7% in *Tragus berteronianus* , and Acid Detergent Lignin (ADL) highest in *Indigofera schimperi* (17.3% ± 0.4%).

For the Mineral Composition, the highest levels of potassium (K) were observed in *Commelina diffusa* (2.6881%), while *Chloris virgata* (1.2385%), *Commelina benghalensis* (1.5826%), and *Cyperus kilimandscharicus* (1.6700%) also exhibited relatively high potassium content. Sodium (Na) concentration was highest in *Chloris ciliaris* (1.0107%) and *Urochloa panicoides* (0.7853%), whereas calcium (Ca) content was notably higher in *Pavonia arabica* (1.1300%) and *Cyperus kilimandscharicus* (0.8150%). Zinc (Zn) levels were highest in *Chloris ciliaris* (0.0027%) and *Commelina diffusa* (0.0026%), while iron (Fe) content was highest in *Dactyloctenium aegyptium* (0.0106%) and *Commelina diffusa* (0.0099%). Copper (Cu) concentration was highest in *Cyperus kilimandscharicus* (0.0011%) and *Indigofera schimperi* (0.0010%). Manganese (Mn) content was highest in *Commelina diffusa* (0.0197%), while magnesium (Mg) levels were highest in *Pavonia arabica* (0.4000%). The statistical analysis indicated significant differences in Zn (*p* = 0.000), K (*p* = 0.000), Mn (*p* = 0.006), Mg (*p* = 0.000), and Ca (*p* = 0.000) concentrations among plant species, while Cu, Fe, Na, and P did not show significant variation (*p* > 0.05) as illustrated in Table S2. Seasons, locations and the plant part consumed (leaf and stem) did not significantly influence the macro‐ or micronutrient levels in the species of plants utilized by hirola antelopes (*p* > 0.05).

### 3.4 Spatial Variations in Chemical Composition of Hirola Forage

**FIGURE 3:**
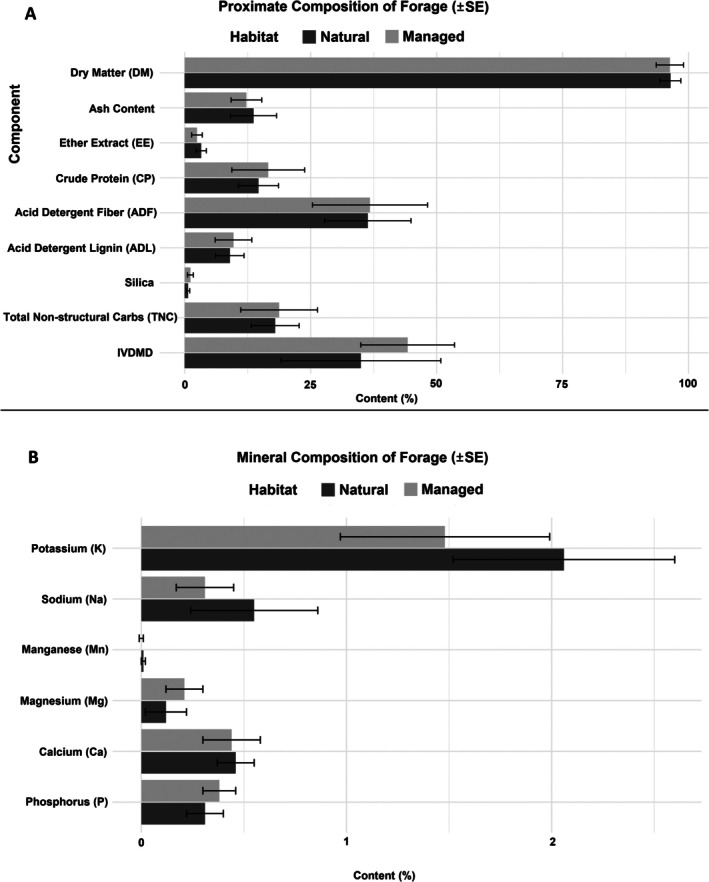
Chemical composition of forage from natural (*n* = 36) and managed (*n* = 40) habitats. Significant differences were observed in selected minerals (*p* < 0.05), with higher Na, K, and Mn in the managed habitat, while Mg and P were higher in the natural habitat. %IVDMD was also significantly greater in the natural habitat. Error bars represent ±1 standard error. See Table S2 for detailed statistical comparisons.

The chemical composition of forage collected from the natural habitat and managed habitat is summarized in Figure 3. Statistical analysis revealed no significant differences in most parameters, including wet weight (*p* = 0.25), dry weight (*p* = 0.46), dry matter (*p* = 0.85), ash (*p* = 0.58), ether extract (*p* = 0.517), crude protein (*p* = 0.265), acid detergent fiber (*p* = 0.884), and acid detergent lignin (*p* = 0.744). However, significant differences were observed in selected mineral components. For example, zinc (0.0005% ± 0.0003%, *F* = 3.154, *p* = 0.000), potassium (1.3064% ± 0.2233%, *F* = 4.75, *p* = 0.000), manganese (0.0073% ± 0.0021%, *F* = 2.425, *p* = 0.006), magnesium (0.2038% ± 0.0425%, *F* = 3.199, *p* = 0.000), and calcium (0.4604% ± 0.1051%, *F* = 4.552, *p* = 0.000) showed notable variation. Species such as *Commelina diffusa* had the highest potassium content (2.6881% ± 0.0231%), while *Pavaniaa arabic*a contained the highest calcium levels (1.1300% ± 0.0000%). Other elements, including copper (0.0007% ± 0.0001%, *F* = 0.759, *p* = 0.736), iron (0.0054% ± 0.0028%, *F* = 0.176, *p* = 1.000), sodium (0.4150% ± 0.0950%, *F* = 1.684, *p* = 0.070), and phosphorus (0.3521% ± 0.0198%, *F* = 0.986, *p* = 0.488) did not exhibit statistically significant differences (*p* > 0.05), suggesting a more uniform distribution across plant species (Table S3).

The sodium (Na) concentration was significantly higher in forage from the managed habitat (0.55% ± 0.31%) compared to the natural habitat (0.31% ± 0.14%; *F*_0.05(1,45)_ = 11.572, *p* = 0.001). Similarly, manganese (Mn) was significantly higher in the managed habitat (0.01% ± 0.01%) compared to the natural habitat (0.00% ± 0.01%; *F*_0.05(1,45)_ = 8.262, *p* = 0.006). Magnesium (Mg) concentration was higher in the natural habitat (0.21% ± 0.09%) compared to the managed habitat (0.12% ± 0.10%), with a significant difference (*F*_0.05(1,45)_ = 9.382, *p* = 0.004). Phosphorus (P) content also showed a significant difference, with higher values in the natural habitat (0.38% ± 0.08%) than in the managed habitat (0.31% ± 0.09%; *F*_0.05(1,45)_ = 7.802, *p* = 0.008). Zinc (Zn) concentration exhibited an extremely significant difference (*F*_0.05(1,45)_ = 42.815, *p* < 0.001), though its absolute values were negligible in both areas. The in vitro dry matter digestibility (%IVDMD) was significantly higher in forage from the natural habitat (44.25% ± 9.30%) compared to the managed habitat (34.97% ± 15.86%), with a statistically significant difference (*F*_0.05(1,45)_ = 5.854, *p* = 0.02) as illustrated in Figure 3.

### 3.5 Seasonal Variation in Nutrient and Mineral Composition

The seasonal variation in mineral composition was not significant for most minerals, including copper (Cu), iron (Fe), zinc (Zn), potassium (K), sodium (Na), manganese (Mn), and phosphorus (P). However, magnesium (Mg) and calcium (Ca) exhibited significant seasonal differences. Magnesium levels were higher during the long rains (0.19% ± 0.11%) compared to the short rains (0.12% ± 0.09%), with a statistically significant difference (*F*_0.05(1,45)_ = 4.053, *p* = 0.050). Similarly, calcium concentrations were greater in the long rains (0.29% ± 0.27%) than in the short rains (0.14% ± 0.15%), with a significant difference (*F*_0.05(1,45)_ = 4.186, *p* = 0.047). In contrast, other minerals, such as phosphorus (*F*_0.05(1,45)_ = 0.037, *p* = 0.849) and silica (*F*_0.05(1,45)_ = 0.021, *p* = 0.885), showed no significant seasonal variation (Table 2).

**TABLE 2:** Seasonal variation in nutrient and mineral composition.

Category of food	Nutritive components	Long rains	Short rains	*F*	Sig.
Proximate composition and fiber fractions	Wet WT	154.67 ± 78.11^a^	147.83 ± 63.95^a^	0.090	0.765
	Dry WT	48.12 ± 17.53^a^	46.56 ± 19.41^a^	0.077	0.783
	%DM	96.22 ± 2.6^a^	96.61 ± 1.90^a^	0.278	0.600
	%Ash	14.25 ± 4.15^a^	13.20 ± 2.60^a^	0.846	0.363
	%EE	3.04 ± 3.45^a^	1.41 ± 0.89^ab^	3.415	0.071
	%CP	14.83 ± 5.63^a^	17.03 ± 6.18^ab^	1.494	0.228
	%ADF	36.65 ± 11.47^a^	36.37 ± 6.70^a^	0.008	0.929
	%ADL	10.82 ± 8.20^a^	11.46 ± 7.36^a^	0.069	0.794
	%Silica	10.80 ± 9.42^a^	11.22 ± 9.23^a^	0.021	0.885
	TNC	4.01 ± 4.20^a^	3.59 ± 2.13^a^	0.138	0.712
	%IVDMD	40.15 ± 12.70^a^	38.61 ± 15.77^a^	0.130	0.721
Mineral composition	Cu %	< 0.01 ± 0.00^a^	< 0.01 ± 0.00^a^	0.011	0.917
	Fe %	< 0.01 ± 0.01^a^	0.01 ± 0.02^ab^	2.707	0.107
	Zn%	< 0.01 ± 0.00^a^	< 0.01 ± 0.00^ab^	3.379	0.073
	K%	1.15 ± 0.45^a^	1.13 ± 0.59^a^	0.018	0.893
	Na%	0.40 ± 0.21^a^	0.49 ± 0.34^a^	1.156	0.288
	Mn%	0.01 ± 0.01^a^	0.01 ± 0.01^a^	0.056	0.813
	Mg%	0.19 ± 0.11^a^	0.12 ± 0.09^b^	4.053	0.050
	Ca%	0.29 ± 0.27^a^	0.14 ± 0.15^b^	4.186	0.047
	P%	0.35 ± 0.10^a^	0.34 ± 0.08^a^	0.037	0.849

*Note:* Values are presented as mean ± standard deviation. Different superscript letters (a, b) within the same row indicate statistically significant differences between seasons at *p* < 0.05. Values sharing the same superscript letter (e.g., both marked “a”) do not differ significantly. *F* and Sig. values represent the *F*‐statistic and significance level from ANOVA, respectively.

Abbreviations: %ADF, Acid Detergent Fiber; %ADL, Acid Detergent Lignin; %Ash, Ash content; %CP, Crude Protein; %DM, Dry Matter; %EE, Ether Extract; %IVDMD, In Vitro Dry Matter Digestibility; %Silica, Silica content; Ca, Calcium; Cu, Copper; Fe, Iron; K, Potassium; Mg, Magnesium; Mn, Manganese; Na, Sodium; P, Phosphorus; TNC, Total Non‐structural Carbohydrates; Zn, Zinc.

### 3.6 Principal Component Analysis of Nutritive Components of Hirola Food

The first five principal components explained 89.2% of the total variance in forage nutritional data, with PC1 accounting for 34.6% and PC2 for 18.2% of the variation (Table 3). These two principal components explained over half (52.8%) of the total variance and captured the major contrasts in forage nutritional composition between habitat types.

**TABLE 3:** Eigenvalues and percentage of variance explained by the first five principal components derived from PCA of forage nutritional composition across hirola habitats. PC1 and PC2 together account for 52.87% of the total variance, capturing major gradients in nutrient quality and digestibility.

Component	Eigenvalue	% Variance	Cumulative %
PC1	4.85	34.62%	34.62%
PC2	2.55	18.24%	52.87%
PC3	2.16	15.46%	68.33%
PC4	1.80	12.83%	81.15%
PC5	1.12	8.02%	89.17%

**FIGURE 4:**
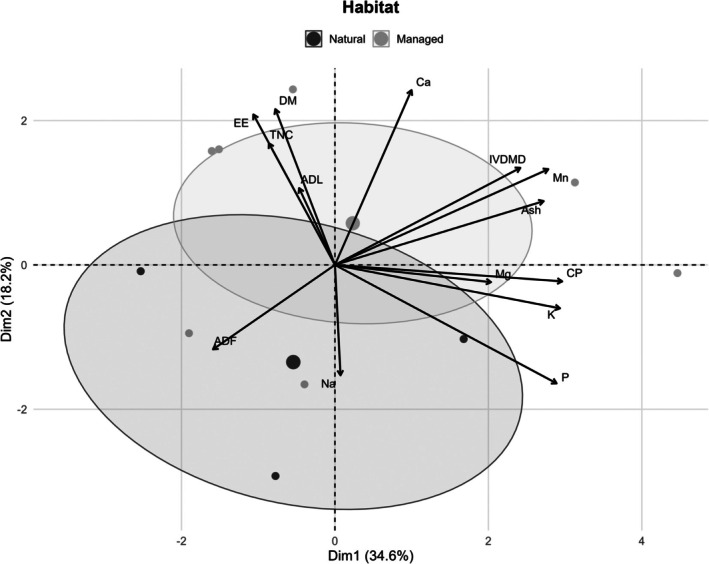
The PCA biplot illustrates the nutritional composition of forage in hirola habitats. The points represent individual samples, the 95% confidence ellipses summarize habitat‐level variation while arrows indicate the direction and strength of each nutrient's contribution to the first two principal components (PC1 and PC2). The biplot reveals differing forage nutritional profiles by habitat type, with Managed habitats having higher concentrations of CP, ash, and minerals, while Natural habitats are characterized by greater ADF and lignin content.

Figure 4 reveals a clear separation of forage samples between the Managed and Natural habitats. While samples from the Managed habitat predominantly clustered within the positive space of PC1, the distribution of samples from the Natural habitat was towards the negative and central regions of the biplot. In addition, there was minimal overlap between the ellipses indicating distinct nutritional profiles between the two habitats. PC1 was driven by variables representing forage nutrient richness, including crude protein (CP; 15.4%), ash (13.0%), TNC (9.9%), Na (9.0%), and ADF (4.4%). On the other hand, PC2 was influenced by variables representing forage digestibility and energy availability such as dry matter (DM; 15.5%), ether extract (EE; 14.5%), and IVDMD (11.3%).

## 4 Discussion

The diet of hirola was mainly composed of grasses in managed habitats, especially during the wet season, while herbs were more prevalent in natural habitats during the dry season. These differences were influenced by grazing intensity, soil conditions, and conservation interventions. Managed habitats, with controlled grazing and habitat restoration, promoted grass cover, whereas natural habitats, experiencing variable grazing pressures, supported a higher abundance of herbs. The shift in plant community composition between managed and natural habitats may have direct implications for the release of hirola from managed conservation areas into natural ecosystems. Hirola in managed habitats have access to a more stable and nutritionally balanced forage base, as controlled grazing minimizes resource depletion and supports plant recovery. This stability contrasts with natural habitats, where the higher prevalence of herbs suggests greater grazing pressure and resource competition, potentially leading to seasonal nutritional deficiencies (Odadi et al. 2011). Such seasonal constraints are particularly consequential for income‐breeding herbivores like hirola whose reproductive success depends on concurrent forage quality rather than stored reserves (Kerby and Post 2013).

Despite these observed trends, our results indicate that plant form distribution differences between habitats and seasons are not significantly pronounced. However, subtle shifts in vegetation composition may still impact hirola foraging behaviors, as past research suggests that changes in plant community structure influence herbivore dietary patterns (Owen‐Smith 2002). The dominance of herbs in natural habitats could force hirola to expand their forage selection to compensate for the reduced availability of preferred grasses, potentially affecting their overall nutritional intake and fitness (Nyman 2010). Similar dietary shifts have been observed in other endangered herbivores, such as the Arabian oryx ( *Oryx leucoryx* ), which demonstrated altered foraging behaviors when translocated from managed reserves to natural desert environments (Strauss 2006). A key finding from our study shows that while overall plant diversity was higher in managed habitats, hirola dietary preferences remained stable across habitat types and seasons. The consistency in dietary choices indicates that hirola selectively forage for high‐nutrient plant species, such as *Chloris virgata* , *Commelina benghalensis* , and *Combretum molle*, irrespective of habitat conditions. The high consumption of *Sporobolus helvolus* despite its neutral selectivity value underscores the role of resource availability in shaping diet composition, independent of nutritional preference. However, due to the lower potassium and sodium levels in the natural habitat, hirola in these areas may experience mineral deficiencies over time. This highlights the need to ensure the availability of these preferred species in both managed and natural habitats to bolster hirola numbers.

Hirola in managed habitats may benefit from conservation practices such as controlled burning and grazing exclusion zones, which enhance forage quality and nutritional composition over time (Sensenig et al. 2010). Without such interventions, natural habitats may exhibit lower forage stability, increasing hirola's reliance on less preferred plant species. The presence of low‐palatable or high‐fiber plants such as *Sida hirta* and *Digitaria ciliaris* in natural habitats may further constrain dietary options, impacting digestion efficiency and overall health. Considering hirola populations in both natural and managed habitats and the release to natural habitats, it is critical to assess how nutritional differences between managed and natural habitats may impact their adaptation and survival. Studies on other large herbivores, such as the European bison ( *Bison bonasus* ) and black rhinoceros ( *Diceros bicornis* ), indicate that translocation success is often tied to the availability of preferred forage species in the release sites (Hofman‐Kamińska and Kowalczyk 2012; Owen‐Smith and Festa‐Bianchet 2003). Our findings indicate that natural habitats were more closely associated with higher levels of phosphorus, magnesium, and in vitro dry matter digestibility (IVDMD), whereas managed habitats were more strongly associated with higher sodium and potassium concentrations. These nutrient gradients reflect spatially structured nutritional landscapes that influence herbivore foraging decisions and movement, as demonstrated for large herbivores in heterogeneous environments (Jesmer et al. 2021).

The separation of forage samples observed in the PCA analysis aligns with ecological expectations for semi‐arid rangelands managed under contrasting grazing regimes. Higher loadings of sodium, potassium, and crude protein in forage from the managed habitat are consistent with reduced grazing pressure and altered nutrient cycling in fenced systems, whereas higher magnesium, phosphorus, and digestibility in the natural habitat likely reflect differences in soil chemistry, species composition, and regrowth dynamics under open grazing conditions. Importantly, the PCA should be interpreted as a descriptive summary of multivariate nutritional variation rather than evidence of adaptive responses or mechanistic processes. These nutritional differences may have direct implications for hirola's dietary intake and overall health. Given the lower potassium and sodium concentrations in forage from natural habitats, short‐term mineral supplementation may be considered to support hirola during transitional phases following release from managed systems. Supplementation, if applied, should be limited to sodium‐ and potassium‐rich mineral licks or salt blocks placed strategically within release areas, rather than direct feeding, to minimize interference with natural foraging behavior. Such supplementation should be implemented only during the initial post‐release period (e.g., the first dry season) and phased out once animals have acclimatized to local forage conditions. Prolonged or indiscriminate supplementation may pose ecological risks, including behavioral dependence, altered movement patterns, and potential disruption of gut microbiota through sustained intake of non‐natural nutrient concentrations (Smit et al. 2007; Hayward and Kerley 2009). We therefore emphasize that supplementation should complement, not replace, habitat restoration efforts aimed at increasing the availability of nutritionally adequate and preferred forage species. Additionally, introducing hay or forage from regions with higher concentrations of these essential minerals may further enhance dietary quality. If hirola are to be successfully relocated into natural habitats, targeted habitat restoration efforts must precede relocation to ensure the presence of nutritionally adequate plant species (Hejcmanová et al. 2019).

To support hirola populations and ensure successful transitions from managed to natural habitats, habitat restoration efforts might prioritize the availability of high‐nutrient plant species. Given that grasses such as *Sporobolus helvolus* and *Chloris virgata* are preferred by hirola, restoration programs could consider focusing on increasing the abundance of these species in natural habitats. Additionally, species like *Pavonia arabica*, which has a high ether extract (EE) content and serves as an important energy source, might be included in restoration efforts to support hirola's energy demands during periods of high expenditure. Considering the significant differences in micronutrient content among plant species, a diverse mix of grasses and forbs should be integrated into restoration initiatives to provide essential micronutrients throughout different seasons. Restoration that enhances spatial clustering of complementary nutrients may improve foraging efficiency and reduce movement costs, consistent with the nutritional landscape framework (Jesmer et al. 2021). Seasonal variations in nutrient availability must be accounted for to ensure that hirola can adjust their diet to meet their physiological needs year‐round.

The PCA results provide specific guidance for habitat enhancement strategies. PC1, which captured variation in crude protein, ash, and mineral content (particularly sodium and potassium), indicates that restoration efforts in natural habitats should prioritize species that contribute to mineral and protein availability while remaining palatable to hirola (Augustine and McNaughton 1998; Grant and Scholes 2006). Highly preferred forbs such as *Commelina benghalensis* and *Commelina diffusa* align with this axis by combining palatability with essential mineral contributions, consistent with forage selection patterns documented in African grazing ungulates (Treydte et al. 2007). Species such as *Indigofera schimperi* , although avoided by hirola due to secondary compounds (Dziba et al. 2006), may still play an indirect role in improving soil nitrogen availability and supporting broader forage quality where appropriately managed (Snyman 2004; Throop and Archer 2008). PC2, associated with digestibility and energy‐related traits (IVDMD, dry matter, and ether extract), suggests that natural habitats already possess an advantage in terms of forage digestibility. This advantage can be enhanced by promoting energy‐rich and moderately preferred species such as *Pavonia arabica*, which combines high ether extract content with appreciable calcium levels, making it particularly valuable during energetically demanding periods such as lactation (Parker et al. 2009; Barboza et al. 2009). Additionally, increasing the abundance of *Chloris virgata* , which exhibited both high selectivity and moderate potassium content, would simultaneously enhance palatability and mineral nutrition (Owen‐Smith and Novellie 1982). The sedge *Cyperus kilimandscharicus*, which showed moderate preference and contributes calcium and trace minerals, could be established near seasonal water sources where soil moisture supports its persistence (Redfern et al. 2003; Smit et al. 2007). By targeting species that align with both PCA axes, balancing nutrient richness with digestibility, restoration programs can better bridge the nutritional gap between managed and natural habitats. The prioritization of these species is further supported by recent ecological niche modeling studies demonstrating their broad climatic tolerance and suitability for restoration under future climate scenarios in semi‐arid East African rangelands (Ali and Muriuki 2025).

Prior to hirola release, potential reintroduction sites should be restored with potassium‐ and sodium‐rich grass species while carefully assessing the risk of dietary shifts due to food supplementation, which may pose challenges for future reintroductions. Animals accustomed to nutrient‐rich supplementation may experience nutritional imbalances after release into natural ecosystems, potentially leading to maladaptive foraging behaviors, reduced survival, and compromised fitness (Teixeira et al. 2007; Wimberger et al. 2009; Berger‐Tal et al. 2020). To mitigate this, we recommend ensuring sufficient natural forage resources to reduce reliance on supplementation and maintain natural foraging behaviors. This can be guided by modeling grass species with the best potential for landscape‐level restoration under current and future climatic conditions, an approach increasingly used to guide climate‐smart rangeland rehabilitation and species prioritization in semi‐arid systems (Ali and Muriuki 2025). Furthermore, long‐term vegetation monitoring, movement tracking, and nutritional analysis are essential to assess the success of hirola relocation and post release as well as habitat restoration efforts. By implementing adaptive management strategies and ensuring access to essential nutrients, conservation efforts can improve habitat suitability and support the long‐term survival of hirola in both managed and natural ecosystems. Conservationists should implement adaptive management strategies that respond to shifting ecological conditions and herbivore dietary preferences (Payne and Bro‐Jørgensen 2016).

In conclusion, the release of hirola from managed to natural habitats presents challenges related to nutritional differences between these management regimes. While managed habitats provide stable and nutritionally diverse forage, natural habitats may lack sufficient high‐quality plant species, necessitating targeted restoration efforts. In addition to restoring key forage species, supplementary feeding measures, such as mineral licks or nutrient‐enriched hay, could be considered to address potential deficiencies. Conservation strategies might prioritize the availability of preferred plant species, ensure habitat heterogeneity, and implement adaptive management practices to support hirola populations effectively. Through these measures, the long‐term survival and population growth of hirola could be achieved.

## Author Contributions

**Abdullahi H. Ali:** conceptualization (equal), data curation (equal), formal analysis (equal), funding acquisition (equal), investigation (equal), methodology (equal), project administration (equal), resources (equal), software (equal), supervision (equal), validation (equal), visualization (equal), writing – original draft (equal), writing – review and editing (equal). **S. Kivai:** conceptualization (equal), data curation (equal), formal analysis (equal), funding acquisition (equal), investigation (equal), methodology (equal), project administration (equal), resources (equal), software (equal), supervision (equal), validation (equal), visualization (equal), writing – original draft (equal), writing – review and editing (equal).

## Conflicts of Interest

The authors declare no conflicts of interest.

## Supporting information


**Table S1:** Plant species utilized by hirola.
**Table S2:** Chemical variation of hirola diet in natural and managed habitats.
**Table S3:** Micronutrients in plant species utilized as food resource for hirola antelope.
**Figure S1:** Variations in plant form distribution across habitat management and seasons.
**Figure S2:** Proportions of plant species utilized by hirola antelopes categorized in their respective forms.

## Data Availability

All the required data are uploaded as Supporting Information.
