# Supplementary material for: Drivers of Hirola Antelope Diet Selection in Natural and Managed Habitat in Eastern Kenya
Source: Ecol Evol. 2026 Jan 22;16(1):e72980. doi: 10.1002/ece3.72980 (PMC12828064; doi:10.1002/ece3.72980)
Supplement: Supplementary file 1 — Table S1: Plant species utilized by hirola. Table S2: Chemical variation of hirola diet in natural and managed habitats. Table S3: Micronutrients in plant species utilized as food resource for hirola antelope. Figure S1: Variations in plant form distribution across habitat management and seasons. Figure S2: Proportions of plant species utilized by hirola antelopes categorized in their respective forms. [file ECE3-16-e72980-s001.docx]

**Supporting Information**

**Table S1: Plant species utilized by hirola**

| Family name | Genera | Species |
| --- | --- | --- |
| Asteraceae | Barleria | *Barleria acanthoides* |
| Combretaceae | Combretum | *Combretum molle* |
| Commelinaceae | Commelina | *Commelina benghalensis* |
|  |  | *Commelina diffusa* |
| Cyperaceae | Cyperus | *Cyperus esculentus* |
|  |  | *Cyperus kilimandscharicus* |
| Fabaceae | Indigofera | *Indigofera schimperi* |
| Malvaceae | Sida | *Sida hirta* |
|  | Pavonia | *Pavonia arabica* |
| Poaceae | Chloris | *Chloris virgata* |
|  |  | *Chloris ciliaris* |
|  | Dactyloctenium | *Dactyloctenium aegyptium* |
|  | Digitaria | *Digitaria ciliaris* |
|  | Eragrostis | *Eragrostis biflora* |
|  | Urochloa | *Urochloa panicoides* |
|  | Sporobolus | *Sporobolus helvolus* |
|  | Tragus | *Tragus berteronianus* |

**Table S2: Chemical variation of Hirola diet in natural and managed habitats**

| Category of food | Nutritive components | Natural habitat | Managed habitat | F | Sig. |
| --- | --- | --- | --- | --- | --- |
| Proximate Composition and Fiber Fractions | Wet WT | 139.83±66.21^a^ | 164.75±78.39^ab^ | 1.356 | 0.250 |
|  | Dry WT | 45.59±17.47^a^ | 49.57±18.69^a^ | 0.555 | 0.460 |
|  | %DM | 96.28±2.70^a^ | 96.42±2.05^a^ | 0.037 | 0.848 |
|  | %Ash | 13.58±3.96^a^ | 14.19±3.46^a^ | 0.311 | 0.580 |
|  | %EE | 2.19±2.56^a^ | 2.76±3.28^a^ | 0.426 | 0.517 |
|  | %CP | 16.57±7.23^a^ | 14.63±3.98^a^ | 1.273 | 0.265 |
|  | %ADF | 36.77±11.42^a^ | 36.34±8.57^a^ | 0.021 | 0.884 |
|  | %ADL | 11.42±10.18^a^ | 10.66±4.67^a^ | 0.108 | 0.744 |
|  | %Silica | 11.90±10.66^a^ | 9.99±7.70^a^ | 0.488 | 0.489 |
|  | TNC | 3.60±4.47^a^ | 4.13±2.50^a^ | 0.246 | 0.622 |
|  | %IVDMD | 44.25±9.30^a^ | 34.97±15.86^b^ | 5.854 | 0.020 |
| Mineral Composition | Cu % | 0.00±0.00^a^ | 0.00±0.00^a^ | 1.588 | 0.214 |
|  | Fe % | 0.01±0.02^a^ | 0.01±0.00^a^ | 0.693 | 0.410 |
|  | %Zn | 0.00±0.00^a^ | 0.00±0.00^b^ | 42.815 | <0.001 |
|  | k% | 1.11±0.49^a^ | 1.17±0.51^a^ | 0.192 | 0.664 |
|  | Na% | 0.31±0.14^a^ | 0.55±0.31^b^ | 11.572 | 0.001 |
|  | Mn (%) | 0.00±0.01^a^ | 0.01±0.01^b^ | 8.262 | 0.006 |
|  | Mg% | 0.21±0.09^a^ | 0.12±0.10^b^ | 9.382 | 0.004 |
|  | Ca% | 0.30±0.21^a^ | 0.18±0.27^ab^ | 2.531 | 0.119 |
|  | P% | 0.38±0.08^a^ | 0.31±0.09^b^ | 7.802 | 0.008 |

*Values are presented as mean ± standard deviation. Different superscripts (a, b) within the same row indicate significant differences at p < 0.05. %DM: Dry Matter; %Ash: Ash content; %EE: Ether Extract; %CP: Crude Protein; %ADF: Acid Detergent Fiber; %ADL: Acid Detergent Lignin; Cu: Copper; Fe: Iron; Zn: Zinc; K: Potassium; Na: Sodium; Mn: Manganese; Mg: Magnesium; Ca: Calcium; P: Phosphorus; %Silica: Silica content; TNC: Total Non-structural Carbohydrates; %IVDMD: In Vitro Dry Matter Digestibility. F and Sig. values represent the F-statistic and significance level from ANOVA, respectively.*

**Table S3: Micronutrients in plant species utilized as food resource for hirola antelope**

| species |  | Cu % | Fe % | %Zn | k% | Na% | Mn (%) | Mg% | Ca% | P% |
| --- | --- | --- | --- | --- | --- | --- | --- | --- | --- | --- |
| *Sporobolous Helvorus* | Mean | 0.0007 | 0.0066 | 0.0004 | 0.7108 | 0.3700 | 0.0009 | 0.1121 | 0.1553 | 0.3521 |
|  | S.E | 0.0001 | 0.0054 | 0.0002 | 0.0585 | 0.0336 | 0.0006 | 0.0132 | 0.0207 | 0.0198 |
| *Chrolis Virgata* | Mean | 0.0007 | 0.0046 | 0.0005 | 1.2385 | 0.3967 | 0.0065 | 0.2038 | 0.2051 | 0.3370 |
|  | S.E | 0.0001 | 0.0013 | 0.0003 | 0.1325 | 0.0717 | 0.0021 | 0.0425 | 0.0480 | 0.0348 |
| *Comerina bengalensis* | Mean | 0.0006 | 0.0059 | 0.0005 | 1.5826 | 0.3674 | 0.0116 | 0.2621 | 0.4604 | 0.3883 |
|  | S.E | 0.0001 | 0.0028 | 0.0004 | 0.1473 | 0.0607 | 0.0040 | 0.0336 | 0.1051 | 0.0652 |
| *Berlaria Anchathoides* | Mean | 0.0007 | 0.0051 | 0.0000 | 1.0533 | 0.5733 | 0.0000 | 0.2200 | 0.3267 | 0.4100 |
|  | S.E | 0.0000 | 0.0030 | 0.0000 | 0.2195 | 0.0694 | 0.0000 | 0.0451 | 0.0677 | 0.0231 |
| *comelina Diffusa* | Mean | 0.0009 | 0.0105 | 0.0006 | 1.6300 | 0.3850 | 0.0100 | 0.2750 | 0.5600 | 0.3900 |
|  | S.E | 0.0002 | 0.0058 | 0.0006 | 0.0700 | 0.0950 | 0.0079 | 0.0150 | 0.1000 | 0.0300 |
| *Eragrostis Biflora* | Mean | 0.0007 | 0.0060 | 0.0014 | 0.9722 | 0.4841 | 0.0074 | 0.1225 | 0.1839 | 0.2956 |
|  | S.E | 0.0001 | 0.0015 | 0.0004 | 0.1329 | 0.1081 | 0.0028 | 0.0326 | 0.0581 | 0.0347 |
| *Sedera hirta* | Mean | 0.0004 | 0.0024 | 0.0006 | 0.9350 | 0.2050 | 0.0000 | 0.1400 | 0.3150 | 0.3650 |
|  | S.E | 0.0003 | 0.0024 | 0.0006 | 0.0050 | 0.0150 | 0.0000 | 0.0200 | 0.0750 | 0.0150 |
| *Cyperus Esculentus* | Mean | 0.0006 | 0.0066 | 0.0013 | 1.2941 | 0.5904 | 0.0047 | 0.0948 | 0.1121 | 0.3814 |
|  | S.E | 0.0001 | 0.0020 | 0.0005 | 0.1964 | 0.1645 | 0.0018 | 0.0306 | 0.0478 | 0.0423 |
| *Pavania Arabica* | Mean | 0.0007 | 0.0047 | 0.0000 | 0.7900 | 0.3500 | 0.0000 | 0.4000 | 1.1300 | 0.3000 |
|  | S.E | 0.0000 | 0.0000 | 0.0000 | 0.0000 | 0.0000 | 0.0000 | 0.0000 | 0.0000 | 0.0000 |
| *Combretum Molle* | Mean | 0.0007 | 0.0000 | 0.0032 | 0.6600 | 0.2000 | 0.0079 | 0.3200 | 0.6200 | 0.2100 |
|  | S.E | 0.0000 | 0.0000 | 0.0000 | 0.0000 | 0.0000 | 0.0000 | 0.0000 | 0.0000 | 0.0000 |
| *Indigojera schimperi* | Mean | 0.0010 | 0.0054 | 0.0000 | 0.7000 | 0.4150 | 0.0000 | 0.3300 | 0.8450 | 0.2350 |
|  | S.E | 0.0003 | 0.0006 | 0.0000 | 0.1400 | 0.0550 | 0.0000 | 0.0300 | 0.2050 | 0.0450 |
| *Cyperus kilimandaschus* | Mean | 0.0011 | 0.0051 | 0.0003 | 1.6700 | 0.3350 | 0.0000 | 0.3350 | 0.8150 | 0.4900 |
|  | S.E | 0.0007 | 0.0051 | 0.0003 | 0.2000 | 0.0950 | 0.0000 | 0.1450 | 0.5550 | 0.0100 |
| *Pavonia arabica* | Mean | 0.0008 | 0.0077 | 0.0020 | 1.0030 | 0.3554 | 0.0086 | 0.1785 | 0.4103 | 0.3180 |
|  | S.E | 0.0002 | 0.0021 | 0.0007 | 0.2080 | 0.0518 | 0.0028 | 0.0637 | 0.2104 | 0.1104 |
| *Tragus Berteronianus* | Mean | 0.0003 | 0.0000 | 0.0000 | 0.5500 | 0.1300 | 0.0045 | 0.2800 | 0.5000 | 0.2200 |
|  | S.E | 0.0000 | 0.0000 | 0.0000 | 0.0000 | 0.0000 | 0.0000 | 0.0000 | 0.0000 | 0.0000 |
| *Digitaria Ciliaris* | Mean | 0.0007 | 0.0000 | 0.0005 | 1.4000 | 0.2500 | 0.0000 | 0.2200 | 0.3100 | 0.4400 |
|  | S.E | 0.0000 | 0.0000 | 0.0000 | 0.0000 | 0.0000 | 0.0000 | 0.0000 | 0.0000 | 0.0000 |
| *Dactyloctenium aegyptium* | Mean | 0.0008 | 0.0106 | 0.0025 | 0.6254 | 0.7082 | 0.0104 | 0.0892 | 0.0309 | 0.2150 |
|  | S.E | 0.0003 | 0.0006 | 0.0006 | 0.1809 | 0.0187 | 0.0002 | 0.0404 | 0.0136 | 0.0150 |
| *Urochloa panisoides* | Mean | 0.0009 | 0.0093 | 0.0024 | 1.3039 | 0.7853 | 0.0073 | 0.0485 | 0.0117 | 0.3220 |
|  | S.E | 0.0001 | 0.0009 | 0.0003 | 0.2233 | 0.1963 | 0.0006 | 0.0203 | 0.0016 | 0.0092 |
| *Commelina diffussa* | Mean | 0.0009 | 0.0099 | 0.0026 | 2.6881 | 0.7450 | 0.0197 | 0.1980 | 0.0573 | 0.3100 |
|  | S.E | 0.0002 | 0.0012 | 0.0002 | 0.0231 | 0.0807 | 0.0031 | 0.0153 | 0.0084 | 0.0600 |
| *Chrolis ciliaris* | Mean | 0.0006 | 0.0084 | 0.0027 | 1.3064 | 1.0107 | 0.0101 | 0.0480 | 0.0217 | 0.2200 |
|  | S.E | 0.0000 | 0.0000 | 0.0000 | 0.0000 | 0.0000 | 0.0000 | 0.0000 | 0.0000 | 0.0000 |
|  | F | 0.759 | 0.176 | 3.154 | 4.75 | 1.684 | 2.425 | 3.199 | 4.552 | 0.986 |
|  | Sig. | 0.736 | 1 | 0 | 0 | 0.07 | 0.006 | 0 | 0 | 0.488 |

*Table S3 presents the micronutrient composition of various plant species utilized as food resources by the hirola antelope. The table includes the mean percentage and standard error (S.E) of essential micronutrients such as Copper (Cu), Iron (Fe), Zinc (Zn), Potassium (K), Sodium (Na), Manganese (Mn), Magnesium (Mg), Calcium (Ca), and Phosphorus (P). These micronutrients play a critical role in the nutritional ecology of hirola, influencing their forage selection and dietary sufficiency. The F-values and significance (Sig.) indicate statistical variation across species, with notable differences in Zinc (p < 0.001), Potassium (p < 0.001), Manganese (p = 0.006), Magnesium (p < 0.001), and Calcium (p < 0.001), while other elements exhibit no significant variation across species. This dataset provides insight into the nutritional profile of plant species in the hirola's habitat and its potential impact on their feeding behavior and overall health.*

Fig S1: Variations in Plant Form Distribution Across Habitat Management and Seasons.

Fig S2: Proportions of plant species utilized by hirola antelopes categorized in their respective forms.
